# Changes in publicly and privately funded care in England following a national programme to reduce provision of low-value elective surgery

**DOI:** 10.1093/bjs/znac390

**Published:** 2022-11-28

**Authors:** Michael Anderson

**Affiliations:** Department of Health Policy, London School of Economics and Political Science, London, UK

## Abstract

**Background:**

This study assessed whether there is an association between changes in publicly and privately funded care for procedures classified as low value by the National Health Service (NHS) in England following implementation of the Evidence-Based Intervention (EBI) programme. Category 1 procedures should not be conducted and are no longer reimbursed by the NHS. Category 2 procedures are only reimbursed by the NHS in certain circumstances.

**Methods:**

Changes in volumes of publicly and privately funded procedures per month in 2019–2020 compared with the previous year were analysed in private hospitals and local healthcare markets, and adjusted for volume of procedures and patient case mix including age, sex, co-morbidities, and deprivation. Supplementary analyses focused on the self-pay and insurance funding mechanisms.

**Results:**

There was a statistically significant association between changes in publicly and privately funded care for category 2 procedures at the hospital (−0.19, 95 per cent c.i. −0.25 to −0.12) and local healthcare market level of analysis (−0.24, −0.32 to −0.15). A statistically significant association for category 1 procedures only existed at the hospital level of analysis (−0.19, −0.30 to −0.08). Findings were similar for patients accessing care through self-pay and insurance funding mechanisms.

**Conclusion:**

Stronger associations between changes in publicly and privately funded care for category 2 procedures may exist as they are clinically indicated in certain circumstances. Reductions in publicly funded care were likely a combined result of the EBI programme and growing NHS waiting lists, whereas increases in privately funded care were influenced by both patient and supplier-induced demand.

## Teaser

This study assessed whether there is an association between changes in publicly and privately funded care for procedures classified as low value by the National Health Service (NHS) in England following implementation of the Evidence-Based Intervention (EBI) programme. There was significant evidence of an association between changes in publicly and privately funded care at both the hospital and local healthcare market level of analysis. Reductions in publicly funded care were likely a combined result of the EBI programme and growing NHS waiting lists, whereas increases in privately funded care were influenced by both patient and supplier-induced demand.

## Introduction

Low-value care can be defined as ‘use of an intervention where evidence suggests it confers no or very little benefit for patients, or risk of harm exceeds likely benefit, or, more broadly, the added costs of the intervention do not provide proportional added benefits’^1^. Disinvestment policies, which are designed to minimize low-value care, can improve healthcare quality and produce cost savings that can be reinvested in other parts of the healthcare system. In the UK, there have been several initiatives to disinvest in low-value care, including national ‘do not do’ recommendations^2^, lists of procedures of limited clinical value drawn up by local commissioning bodies^3^, and recommendations by professional societies on inappropriate tests and procedures^4^. There have been several evaluations of these efforts, with mixed results^5–8^, but they have focused exclusively on publicly funded care because of limited available data on privately funded care.

It is important to investigate the dynamic between changes in publicly and privately funded care following the implementation of disinvestment policies for procedures considered as low value by public funders as significant increases in privately funded care may signal unmet need among patients. This is because many procedures considered as low value are beneficial for patients in certain circumstances. Conversely, significant increases in privately funded care may signal inappropriate supplier-induced demand (demand for healthcare that is created by the supplier or provider of healthcare services), particularly if there is limited evidence of clinical effectiveness or cost-effectiveness for the procedure in any circumstance. For private hospitals in England, it is reasonable to assume that there may be some relationship between changes in publicly and privately funded care. This is because private hospitals provide a similar proportion of publicly and privately funded elective care^9,10^. Since the mid-2000s, national efforts to clear waiting lists and promote competition have resulted in private hospitals providing an increasing quantity of publicly funded care^11^. Moreover, there continues to be a significant market for privately funded care, with approximately 10 per cent of the population being covered by some form of private health insurance^12^, and the self-pay market for private healthcare growing by approximately 7 per cent per year between 2010 and 2019^13^.

This study took advantage of two developments to establish whether there is an association between changes in publicly and privately funded care for procedures classified as low value by the National Health Service (NHS) in England. First, the UK Government^14^ published an investigation in 2014 that introduced a mandatory requirement for private hospitals to collect and submit data to the Private Health Information Network (PHIN) from January 2016. This means that for the first time reliable data on hospital activity in the private healthcare sector have been collected in England. Second, the NHS in England has launched a national initiative to disinvest in low-value care called the Evidence-Based Procedures (EBI) programme. The first phase of the EBI programme included 17 procedures (*Table 1*)^15^.

**Table 1:** National Health Service England Evidence-Based Intervention programme procedures

Category	Procedure
Category 1	Intervention for snoring (not OSA)
	Dilatation and curettage for heavy menstrual bleeding
	Knee arthroscopy with osteoarthritis
	Injection for non-specific low back pain without sciatica
Category 2	Breast reduction
	Removal of benign skin lesions
	Grommets
	Tonsillectomy
	Haemorrhoid surgery
	Hysterectomy for heavy bleeding
	Chalazia removal
	Shoulder decompression
	Carpal tunnel syndrome release
	Dupuytren’s contracture release
	Ganglion excision
	Trigger finger release
	Varicose vein surgery

Source National Health Service (NHS) England^15^. OSA, obstructive sleep apnoea.

Category 1 procedures are those that have been shown to be ineffective and should no longer be offered to patients, whereas category 2 procedures are understood to be only clinically appropriate in certain circumstances. The EBI programme involves more than just soft measures such as publishing guidance, and incorporates more restrictive policies such as targets, previous approval processes, and withdrawal of reimbursement for certain procedures. In England, central funds are allocated to local bodies known as Clinical Commissioning Groups (CCGs), which are responsible for commissioning hospital services for their respective local populations. Targets were set for CCGs to reduce the number of category 1 procedures to ‘near zero’, and for category 2 procedures to be reduced to the 25th percentile of the age–sex standardized rate for each CCG. A zero tariff for category 1 procedures was introduced, meaning that hospitals were no longer reimbursed for carrying out these procedures. For category 2 procedures, hospitals are expected to seek prior approval from CCGs with clear clinical justification before providing the procedure. Finally, progress in meeting agreed targets is monitored and benchmarked using a publicly available dashboard^16^.

## Methods

### Study cohort and data sources

The study cohort comprised all private hospitals in England providing 16 of the 17 EBI procedures between 1 April 2017 and 28 February 2020. Data beyond this point were not analysed as elective care activity was significantly influenced by the COVID-19 pandemic. Removal of benign skin lesions was excluded from the analysis as this is a relatively minor procedure that often takes place in outpatient clinics rather than in a surgical theatre, and, as a relatively high-volume procedure, its inclusion could bias results. Data for publicly funded care were retrieved from the Secondary Uses Service (SUS) data set provided by NHS Digital (the non-departmental public body responsible for information, data, and IT systems in England). Data on privately funded care were retrieved from the PHIN, the mandated health information organization responsible for data collection and reporting of activity in the private healthcare sector since January 2016^14^. Data were extracted for individual-patient characteristics including age, sex, deprivation, and co-morbidities. Co-morbidities were coded according to the Charlson Co-morbidity Index (CCI)^17^, and deprivation according to the English Index of Multiple Deprivation (IMD) 2019^18^. The PHIN and SUS data sets record data in finished episodes of care, which relates to the clinician responsible for the respective aspect of care. To avoid counting single procedures multiple times, procedures were identified according to each unique hospital spell rather than the finished consultant episode.

Procedures were identified using different combinations of OPCS-4^19^, and ICD-10^20^ codes. Relevant hospital spells were first extracted using groups of OPCS-4 codes and then inclusion criteria based on ICD-10 codes were applied to reflect indications when each procedure is classified as low value. These combinations of OPCS-4 and ICD-10 codes were developed by the EBI programme based on a literature review and feedback from stakeholders, including CCGs, hospitals, and specialty organizations. These codes are available publicly^15^, and a summary can be found in *Table S1*. To illustrate the impact of using ICD-10 codes to identify EBI procedures, volumes of procedures from the PHIN data set before and after application of the ICD-10 inclusion criteria are reported (*Table S2*). This analysis was not undertaken for the SUS data set as NHS Digital provided an extract of relevant hospital spells after the inclusion criteria had already been applied. As the PHIN data set is relatively newly established, the percentage of hospital spells with an ICD-10 code present for the dominant diagnosis was also reported to establish whether this changed over time (*Table S3*).

### Study outcomes

The primary study outcome was monthly changes in volumes of privately funded category 1 and category 2 EBI procedures between April 2019 and February 2020 compared with the same month in the previous year. To account for potential heterogeneity in trends for individual procedures, all analyses were repeated at the individual-procedure level. As a robustness analysis, the monthly change in volume between April 2019 and February 2020 and the same month 2 years previously was also calculated to account for potential anticipatory change as the commissioning guidance for the EBI programme was published in November 2018^15^.

The primary study outcome was analysed at both the hospital and local healthcare market level of analysis. Hospitals of interest were private hospitals, rather than NHS hospitals, which perform only small volumes of privately funded procedures^9^. Local healthcare markets were defined by changes in volumes of the provision of privately and publicly funded care, including NHS hospitals, within 30 km from any private hospital. This was estimated using the STATA code ‘geonear’^21^, which uses Vincenty’s formulae to calculate the direct distances between two points. The postcode of each hospital was geocoded into longitude and latitude coordinates using a freely available batch geocoding service^22^. Each local healthcare market was not mutually exclusive, meaning that changes in volume in hospitals could be included in multiple local healthcare markets. This definition reflects how NHS and private hospitals function in practice in England as local healthcare markets do not operate independently of one another and typically overlap^23^. This definition of local healthcare markets has been used several times previously in literature focused on competition in local healthcare markets in England^24–26^.

Secondary outcomes included changes in volume of category 1 and 2 EBI procedures accessed through either self-pay or insurance funding mechanisms. This analysis was conducted as changes in volumes of self-pay care are reflective of individual willingness to pay, and therefore potentially more representative of unmet need for NHS care than changes in insurance-funded care. In contrast, changes in volume of insurance-funded care can be influenced by a variety of factors, including unmet need for NHS care, trends in the number of insurance policies nationally, and the coverage policies by individual insurers for specific procedures. As the PHIN data set does not include data on the specific insurer associated with each hospital spell, it was not possible to explore the impact of these different factors on trends for insurance-funded care.

### Statistical analysis

Ordinary least squares (OLS) regression analysis with fixed effects was used to analyse the association between changes in volume of privately funded and publicly funded procedures using Stata^®^ SE version 16 (StataCorp, College Station, TX, USA). All models were adjusted to reflect the total volume of procedures conducted in each hospital, and characteristics of patients treated at each hospital including age, sex, and CCI and IMD scores. The full regression equation is available in *supplementary material* . A fixed-effect model was used as the assumptions of independence and homogeneity of variance, required for simple OLS estimators, were not met as panel data were analysed, meaning that there was correlation between observations over time and within hospitals^27^. To account for this, the fixed-effect estimator differenced out all time-invariant hospital characteristics from the equation, and standard errors were also clustered at the hospital level. Multicollinearity and Hausman assumption tests were applied to ensure that the models were specified correctly (*Tables S4 and S5*)^28,29^. Scatter plot graphs were also produced to ensure that there was no evidence of non-linear trends between the dependent and independent variables (*Figs S1 and S2*).

## Ethical approval

As a service development evaluation based on routinely collected data, further ethics approval from NHS Research Authority was not required in accordance with the NHS Health Research Authority online decision tool, which is based on the UK Policy Framework for Health and Social Care Research. Data were handled in accordance with NHS England information governance policies, and through this study and subsequent write-up, researchers followed the London School of Economics and Political Science Code of Research Conduct. This study has been screened to ensure that no confidential information has been revealed.

## Results

### Descriptive statistics

**Fig. 1:**
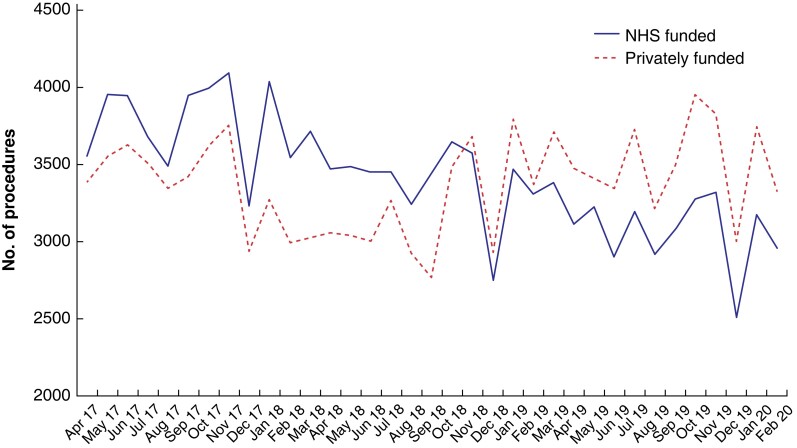
Trends in National Health Service-funded and privately funded Evidence-Based Intervention procedures undertaken in private hospitals between April 2017 and February 2020 NHS, National Health Service.

Analysis of trends in the number of EBI procedures conducted in private hospitals revealed that, from November 2018, the number of privately funded procedures overtook and was consistently above the number of publicly funded procedures (*Fig. 1*). These trends were reflected for most individual procedures when comparing financial years (*Table 2*). There was no evidence that diagnostic coding in the PHIN data set varied significantly between procedures or over time (*Table S3*); the completeness of dominant diagnosis coding was above 95 per cent for all procedures in every financial year.

**Table 2:** Volumes of National Health Service-funded and privately funded Evidence-Based Intervention procedures undertaken in private hospitals in England 2017–2018 to 2019–2020

	2017–2018		2018–2019		2019–2020	
	Privately funded	NHS-funded	Privately funded	NHS-funded	Privately funded	NHS-funded
**Category 1 procedures**	8972	9353	8971	8432	9820	7401
ȃSurgical intervention for snoring (not OSA)	47	56	35	51	45	40
ȃDilatation and curettage for heavy menstrual bleeding	60	88	57	40	75	52
ȃKnee arthroscopy with osteoarthritis	220	1349	339	871	424	763
ȃInjection for non-specific low back pain without sciatica	8645	7860	8540	7470	9276	6546
**Category 2 procedures**	28 485	32 144	26 389	28 899	28 739	26 298
ȃBreast reduction	2630	–*	2701	9	2852	–*
ȃGrommets	355	221	329	230	380	227
ȃTonsillectomy	4051	1750	3945	1688	4007	1264
ȃHaemorrhoid surgery	1399	1680	1356	1655	1359	1611
ȃHysterectomy for heavy bleeding	2910	1793	2820	1728	2906	1693
ȃChalazia removal	134	205	137	188	206	187
ȃShoulder decompression	2867	5309	2446	3795	2563	2613
ȃCarpal tunnel syndrome release	2960	11 384	2988	10 307	3861	9968
ȃDupuytren’s contracture release	869	3625	1056	3710	1223	3583
ȃGanglion excision	623	1926	728	1537	849	1522
ȃTrigger finger release	670	2323	801	2248	868	2061
ȃVaricose vein surgery	9017	1922	7082	1804	7665	1563

These volumes reflect the number of hospital spells that meet the inclusion criteria for Evidence-Based Intervention (EBI) procedures based on ICD-10 codes developed to reflect instances of low-value care. Therefore, the total number of procedures that do not meet these criteria is much higher. March has been removed from the data for all financial years to account for the influence of the emergence of the COVID-19 pandemic. *The Private Health Information Network applies a policy of small number suppression for any activity levels below 8. OSA, obstructive sleep apnoea.

Mean patient characteristics indicated that patients undergoing privately funded EBI procedures were consistently younger, had a smaller number of co-morbidities, and resided in less deprived areas (*Table 3*). A higher proportion of patients undergoing category 1 procedures were women. This aligns with literature indicating that the prevalence of low back pain^30,31^, and knee arthritis^32,33^, is higher in women. Similarly, a higher proportion of patients undergoing category 2 procedures was women. Two category 2 procedures are performed exclusively for women, specifically breast reduction and hysterectomy for heavy bleeding (*Table 2*).

**Table 3:** Patient characteristics for National Health Service-funded and privately funded Evidence-Based Intervention procedures undertaken in private hospitals 2017–2018 to 2019–2020

	2017–2018		2018–2019		2019–2020	
	Privately funded	NHS-funded	Privately funded	NHS-funded	Privately funded	NHS-funded
**Category 1 procedures**						
ȃAge (years)	56.10 (55.76, 56.43)	58.31 (58.00, 58.62)	56.49 (56.16, 56.82)	58.67 (58.34, 59.00)	56.13 (55.81, 56.45)	58.51 (58.16, 58.85)
ȃSex*	0.53 (0.52, 0.54)	0.62 (0.61, 0.63)	0.54 (0.53, 0.55)	0.62 (0.61, 0.63)	0.53 (0.52, 0.54)	0.62 (0.61, 0.63)
ȃCCI score	0.11 (0.10, 0.12)	0.24 (0.23, 0.26)	0.12 (0.11, 0.12)	0.26 (0.25, 0.27)	0.11 (0.10, 0.11)	0.23 (0.22, 0.25)
ȃIMD score	13.69 (13.48, 13.89)	21.04 (20.73, 21.36)	13.28 (13.08, 13.48)	21.33 (21.01, 21.66)	13.31 (13.12, 13.50)	21.62 (21.26, 21.97)
**Category 2 procedures**						
ȃAge (years)	48.61 (48.40, 48.83)	54.98 (54.80, 55.15)	48.82 (48.59, 49.05)	55.10 (54.91, 55.28)	49.07 (48.84, 49.29)	55.76 (55.56, 55.96)
ȃSex*	0.66 (0.66, 0.67)	0.58 (0.58, 0.59)	0.66 (0.65, 0.66)	0.57 (0.57, 0.58)	0.64 (0.64, 0.65)	0.58 (0.58, 0.59)
ȃCCI score	0.08 (0.07, 0.08)	0.20 (0.19, 0.20)	0.09 (0.08, 0.09)	0.21 (0.21, 0.22)	0.09 (0.09, 0.09)	0.21 (0.21, 0.22)
ȃIMD score	13.69 (13.57, 13.81)	19.68 (19.52, 19.84)	13.27 (13.15, 13.39)	19.52 (19.35, 19.69)	13.47 (13.35, 13.59)	19.50 (19.33, 19.68)
**Overall**						
ȃAge (years)	50.40 (50.22, 50.59)	55.73 (55.57, 55.88)	50.77 (50.57, 50.96)	55.91 (55.74, 56.07)	50.86 (50.68, 51.05)	56.36 (56.19, 56.54)
ȃSex*	0.63 (0.63, 0.64)	0.59 (0.59, 0.59)	0.63 (0.62, 0.63)	0.58 (0.58, 0.59)	0.61 (0.61, 0.62)	0.59 (0.58, 0.59)
ȃCCI score	0.09 (0.08, 0.09)	0.21 (0.20, 0.21)	0.09 (0.09, 0.10)	0.22 (0.22, 0.23)	0.09 (0.09, 0.10)	0.22 (0.21, 0.22)
ȃIMD score	13.69 (13.59, 13.79)	19.99 (19.84, 20.13)	13.27 (13.17, 13.37)	19.93 (19.78, 20.08)	13.44 (13.34, 13.54)	19.97 (19.81, 20.13)

Values are mean (95% c.i.). March has been removed from the data for all financial years to account for the influence of the emergence of the COVID-19 pandemic. *Sex data based on score 1 for women and 0 for men. CCI, Charlson Co-morbidity Index; IMD, Index of Multiple Deprivation.

### Regression model

There was a statistically significant association between total changes in publicly and privately funded care at the hospital level of analysis (−0.17, 95 per cent c.i. −0.24 to −0.10), with similar findings for category 1 (−0.19, −0.30 to −0.08) and category 2 (−0.19, −0.25 to −0.12) procedures (*Table 4*). This is approximately equivalent to an increase of one privately funded procedure for every five fewer publicly funded procedures. At the local healthcare market level of analysis, there was a statistically significant association between total changes in publicly and privately funded care (−0.28, −0.41 to −0.15), and for category 2 procedures (−0.24, −0.32 to −0.15) (*Table 5*). This is approximately equivalent to an increase of one privately funded procedure for every four fewer publicly funded procedures. However, this association was not significant for category 1 procedures (−0.11, −0.28 to 0.07). There was no clear pattern for the association between patient characteristics and changes in volumes of privately funded care. However, there was a consistent and positive association between the total volume of procedures conducted in hospitals and increases in privately funded care at both the hospital and local healthcare market level of analysis. This suggests that larger private hospitals had greater increases in privately funded procedures during the period of analysis, which could have been the result of having greater capacity to take advantage of increased demand for EBI procedures than smaller hospitals. These findings were replicated in the sensitivity analysis that examined monthly change in 2019–2020 *versus* 2017–2018 (*Table S6*), although there was also a statistically significant finding for category 1 procedures at the local healthcare market level of analysis (−0.43, −0.84 to −0.02).

**Table 4:** Association between National Health Service-funded and privately funded monthly volume change between 2019–2020 and 2018–2019: hospital analysis

	Overall		Category 1		Category 2	
	Co-efficient	P*	Co-efficient	P*	Co-efficient	P*
Δ NHS volume	−0.17 (−0.24, −0.10)	<0.001	−0.19 (−0.30, −0.08)	<0.001	−0.19 (−0.25, −0.12)	<0.001
Sex	2.71 (−0.81,6.21)	0.130	−1.35 (−4.64, 1.94)	0.417	3.72 (0.61, 6.83)	0.019
CCI score	−0.29 (−4.56, 3.99)	0.895	−6.18 (−9.35, −3.02)	<0.001	−0.99 (−4.82, 2.30)	0.553
Age (years)	−0.02 (−0.14, 0.09)	0.698	0.01 (−0.09, −0.10)	0.908	−0.07 (−0.17, 0.02)	0.118
IMD score	−0.09 (−0.20, 0.03)	0.126	−0.12 (−0.20, −0.03)	0.007	0.00 (−0.09, 0.10)	0.944
Total volume	0.46 (0.39, 0.53)	<0.001	0.53 (0.40, 0.67)	<0.001	0.48 (0.41, 0.55)	<0.001
Constant	−16.12 (−23.63,−8.61)	<0.001	−6.59 (−12.17,−1.01)	0.021	−12.41 (−18.83,−5.99)	<0.001
No. of observations	1336		357		1290	
Adjusted *R*^2^	0.195		0.295		0.193	
No. of hospitals	142		68		141	

Values in parentheses are 95% confidence intervals, March has been removed from the data for all financial years to account for the influence of the emergence of the COVID-19 pandemic. Δ, difference between 2019–2020 and 2018–2019; NHS, National Health Service; CCI, Charlson Co-morbidity Index; IMD, Index of Multiple Deprivation. *P values are derived from ordinary least squares regression used to analyse the association between the above dependent variables and changes in volume of privately funded procedures.

**Table 5:** Association between National Health Service-funded and privately funded monthly volume change between 2019–2020 and 2018–2019: local healthcare market analysis

	Overall		Category 1		Category 2	
	Co-efficient	P*	Co-efficient	P*	Co-efficient	P*
Δ NHS volume	−0.28 (−0.41, −0.15)	<0.001	−0.11 (−0.28, 0.07)	0.230	−0.24 (−0.32, −0.15)	<0.001
Sex	8.10 (−90.53, 106.74)	0.872	−17.44 (−59.21, 24.33)	0.411	−6.63 (−97.21, 83.96)	0.885
CCI score	−27.33 (−113.53, 58.86)	0.553	−31.65 (−64.69, 1.39)	0.060	−20.42 (−91.23, 50.38)	0.570
Age (years)	−2.71 (−4.80, −0.61)	0.012	−0.77 (−2.45, 0.91)	0.366	−2.82 (−4.79, −0.85)	0.005
IMD score	−1.84 (−6.24, 2.57)	0.412	−0.61 (−1.93, 0.71)	0.361	0.10 (−4.30, 4.50)	0.964
Total volume	0.68 (0.44, 0.93)	<0.001	0.82 (0.33, 1.31)	<0.001	0.59 (0.48, 0.71)	<0.001
Constant	−657.86 (−1003.76, −311.96)	<0.001	−426.31 (−714.25, −138.38)	0.004	−370.78 (−575.74, −165.82)	<0.001
No. of observations	2123		1150		2105	
Adjusted *R*^2^	0.353		0.416		0.255	
No. of hospitals	216		164		212	

Values in parentheses are 95% confidence intervals, March has been removed from the data for all financial years to account for the influence of the emergence of the COVID-19 pandemic. Δ, difference between 2019–2020 and 2018–2019; NHS, National Health Service; CCI, Charlson Co-morbidity Index, IMD, Index of Multiple Deprivation. *P values are derived from ordinary least squares regression used to analyse the association between the above dependent variables and changes in volume of privately funded procedures.

Additional supplementary analyses included assessing whether there was an association between changes in publicly and privately funded procedures accessed through either the self-pay or insurance funding mechanisms (*Tables S7–S11*). These models produced similar findings to the primary analysis, with a statistically significant association between changes in publicly and privately funded care at both the hospital and local healthcare market level of analysis, and no statistically significant association for category 1 procedures at the local healthcare market level of analysis.

Focusing on individual category 1 procedures (*Table S12*), there was a statistically significant association between total changes in publicly and privately care at the hospital level of analysis for injections for non-specific low back pain without sciatica for all privately funded care (−0.21, −0.35 to −0.07), insurance-funded care (−0.16, −0.30 to −0.02), and self-paid care (−0.16, −0.26 to −0.06) (*Table 5*). However, there was no statistically significant association at the local healthcare market level of analysis. There were too few observations to produce coefficient estimates for other individual category 1 procedures, with the exception of knee arthritis for osteoarthritis at the local healthcare market level of analysis (−0.32, −0.64 to 0.00). Focusing on individual category 2 procedures, the association between changes in publicly and privately funded care at the hospital level of analysis was the largest for tonsillectomy (−0.38, −0.50 to −0.27) and hysterectomy for heavy bleeding (−0.42, −0.58 to −0.26) (*Table 5*). The association at the local healthcare market level of analysis was largest for grommets (−0.87, −1.03 to −0.72), tonsillectomy (−0.52, −0.75 to −0.30), haemorrhoid surgery (−0.33, −0.41 to −0.25), hysterectomy for heavy bleeding (−0.34, −0.55 to −0.14), and varicose vein surgery (−0.36, −0.49 to −0.23). Findings were similar for patients accessing category 2 procedures through insurance funding mechanisms, but there was only a statistically significant association between changes in publicly funded and self-paid care for varicose vein surgery at the hospital (−0.13, −0.26 to −0.01) and local healthcare market level of analysis (−0.33, −0.49 to −0.16), and tonsillectomy at the hospital level of analysis (−0.17, −0.33 to −0.01).

## Discussion

This study has demonstrated an association between reductions in publicly funded care and increases in privately funded care in private hospitals for procedures classified as low value by the NHS in England following the implementation of the EBI programme. For both category 1 and 2 procedures, this association is approximately equivalent to an increase in one privately funded procedure for every five fewer publicly (NHS) funded procedures conducted. Focusing on local healthcare markets, which also take account of reductions in publicly funded care at NHS hospitals within a 30-km radius of private hospitals, there were mixed results. For category 2 procedures, the association between changes in publicly and privately funded care was approximately equivalent to an increase in one privately funded procedure for every four fewer publicly funded procedures conducted. For some individual procedures, the association was bigger, including for grommets, tonsillectomy, haemorrhoid surgery, hysterectomy for heavy bleeding, and varicose vein surgery. In contrast, there was no statistically significant association between changes in publicly and privately funded care for category 1 procedures at the local healthcare market level of analysis. This may be because category 2 procedures are clinically appropriate in certain circumstances, and it is more plausible that physicians in NHS hospitals would direct patients to access these procedures in the private healthcare sector than for category 1 procedures, which are understood to be not clinically effective or cost-effective in any circumstance. There were no obvious differences between findings for changes in insurance-funded and self-paid care; therefore, the authors have not generated any evidence indicating that unmet need among patients for EBI procedures is more significant than other factors driving increases in privately funded care, such as supplier-induced demand or increases in the number of private health insurance policies.

There are a number of important strengths to this work. First, it is the first analysis from the UK that has explored whether an association exists between changes in publicly funded and privately funded care for procedures undergoing disinvestment by the NHS. Second, although this analysis focused specifically on private hospitals, the impact of changes in volumes of publicly funded care in NHS hospitals was captured through the local healthcare market analysis. Third, through additional supplementary analysis, this study explored changes in the volume of procedures accessed through different financial mechanisms, specifically accessed through either insurance or out-of-pocket payments.

However, there are also potential limitations associated with this analysis. Only data for a limited time interval were analysed. This is because the PHIN has only collected data on privately funded care since January 2016, and it was not possible to conduct analyses beyond February 2020 because of the COVID-19 pandemic. Second, there are limitations in using NHS Digital and PHIN data sets to identify procedures as relevant codes are typically determined by clinical coders who work from patient notes and have little contact with frontline clinical staff. Consequently, misinterpretations and omissions can occur^34,35^. Moreover, the PHIN data set is newly established and has been used less for research purposes than NHS Digital data sets^36^. However, this study has demonstrated that the quality of diagnostic coding in the PHIN data set is high and did not change significantly over time during the period of analysis of this study. Third, there is potential for reverse causation in this analysis. Specifically, increased provision of privately funded care may result in reductions in publicly funded care, rather than vice versa. However, this is unlikely as trends in elective care provision were analysed following the implementation of a national policy that actively sought to reduce publicly funded care for specific procedures. Fourth, it was not possible to analyse data at the physician level of analysis as consistent identifiers were not available across PHIN and NHS Digital data sets. As most hospital consultants working in private hospitals also hold contracts in NHS hospitals, analysing changes in volume at the physician level of analysis would have potentially identified strong evidence of supplier-induced demand. Finally, this study cannot disentangle the impact of the several concurrent changes that were happening during the period of analysis. NHS waiting lists to access publicly funded care grew from 3.8 million in April 2017 to 4.4 million in February 2020^37^, and hospitals may have deprioritized procedures understood to be of low value in favour of more urgent and high-value procedures, even without the EBI programme. There was also a slight expansion of the number of private health insurance policies in the UK from 3.98 to 4.10 million between 2017 and 2020^38^, which may have contributed to increases in privately funded care. Conversely, some insurers may have anticipated the impact of the EBI programme and tightened coverage policies to restrict access to certain procedures. Hospitals or physicians may have also engaged with selective coding of diagnoses to avoid procedures being classified as low value, potentially influencing trends in both publicly and private funded care. Therefore, this study cannot demonstrate the causal impact of the EBI programme and instead analysed only the relative association between changes in publicly and privately funded care while acknowledging that multiple factors were driving these trends.

This study has demonstrated reductions in publicly funded care associated with increases in privately funded care following a national initiative to reduce the provision of procedures classified as low value in certain circumstances. However, the extent to which this increased demand in the private healthcare sector has been driven by patients or suppliers cannot be stated conclusively. Therefore, further qualitative and operational research is needed to gain a more complete understanding of the associations in this study. This will involve mapping referral pathways for patients accessing privately funded care, and structured interviews to ascertain the reasons underlying decisions to seek care outside the NHS. Further investigation is also needed to establish whether the target set by the NHS in England to reduce provision of EBI procedures to 25 per cent of baseline levels is appropriate or evidence-based for all procedures. There is also scope to develop more appropriate region-specific targets that reflect disease epidemiology rather than just the age and sex of local populations. Moving forward, this study has highlighted a need for greater collaboration between the NHS, private providers, and insurers to ensure a coordinated response to disinvestment in low-value care, including the potential risk of unmet need for healthcare when these procedures are indicated clinically.

## Supplementary Material

Znac390_Supplementary_Data

## Data Availability

The controller of the data analysed on publicly funded care is NHS Digital, whereas the controller of data analysed on privately funded care is the PHIN. Patient-level data are available from NHS Digital and PHIN, subject to their information governance requirements.
