# Supplementary material for: Changes in publicly and privately funded care in England following a national programme to reduce provision of low-value elective surgery
Source: Br J Surg. 2022 Nov 28;110(2):209–16. doi: 10.1093/bjs/znac390 (PMC10364485; doi:10.1093/bjs/znac390)
Supplement: Znac390_Supplementary_Data [file znac390_supplementary_data.docx]

***Supplementary Material***

**Title:** **Moving from guidelines to more restrictive disinvestment policies: implications for public and private elective care provision in England**

Michael Anderson^1^

1. Department of Health Policy, The London School of Economics and Political Science, London, WC2A 2AE, United Kingdom

**Corresponding author.** Michael Anderson. **ORCID ID**; <https://orcid.org/0000-0002-8454-4640>

**Contents**

[1. Supplementary Methods 3](#_Toc119937773)

[1.1. Procedure and diagnostic code inclusion criteria 3](#_Toc119937774)

[Table 1: Procedure and diagnostic codes for Evidence Based Interventions (EBI) procedures 3](#_Toc119937775)

[Table 2: Privately funded Evidence Based Interventions (EBI) procedures in private hospitals in before and after application of diagnostic code inclusion criteria ^a^ 4](#_Toc119937776)

[Table 3: Completeness of dominant diagnosis coding for privately funded Evidence Based Interventions (EBI) procedures in private hospitals in England 2017/18 to 2019/20 ^a^ 5](#_Toc119937777)

[1.2. Regression Model Equation 6](#_Toc119937778)

[1.3. Assumption and Model Specification tests 7](#_Toc119937779)

[Table 4: Multicollinearity test for association between NHS and privately funded monthly volume change between 2019/20 and 2018/19 for Evidence Based Interventions (EBI) procedures^a^ 7](#_Toc119937780)

[Table 5: Model specification tests for association between NHS and privately funded monthly volume change for Evidence Based Interventions (EBI) procedures 7](#_Toc119937781)

[Figure 1: Scatter plot graph of observations for dependent and independent variables (Hospital analysis) 8](#_Toc119937782)

[Figure 2: Scatter plot graph of observations for dependent and independent variables (Local healthcare market analysis) 9](#_Toc119937783)

[2. Supplementary Results 10](#_Toc119937784)

[Table 6: Association between NHS and Private monthly volume change between 2019/20 and 2017/18 ^a^ 10](#_Toc119937785)

[Table 7: Volumes of NHS, insured, and self-pay funded Evidence Based Interventions (EBI) procedures in private hospitals in England 2017/18 to 2019/20 ^a^ 11](#_Toc119937786)

[Table 8: Association between NHS and privately funded monthly volume change between 2019/20 and 2018/19 (Insurance analysis) ^a^ 12](#_Toc119937787)

[Table 9: Association between NHS and privately funded monthly volume change between 2019/20 and 2018/19 (Self pay analysis) ^a^ 13](#_Toc119937788)

[Table 10: Association between NHS and privately funded monthly volume change between 2019/20 and 2017/18 (Insurance analysis) ^a^ 14](#_Toc119937789)

[Table 11: Association between NHS and privately funded monthly volume change between 2019/20 and 2017/18 (Self pay analysis) ^a^ 15](#_Toc119937790)

[Table 12: Association between NHS and privately funded monthly volume change between 2019/20 and 2018/19 for individual Evidence Based Interventions (EBI) procedures ^a^ 16](#_Toc119937791)

[3. References 17](#_Toc119937792)

# Supplementary Methods

## Procedure and diagnostic code inclusion criteria

### Table 1: Procedure and diagnostic codes for Evidence Based Interventions (EBI) procedures

| Procedure | OPCS codes | Diagnostic codes |
| --- | --- | --- |
| Intervention for snoring (not OSA) | F324, F325, F326, Y114, F328 | G473* |
| Dilatation & curettage for heavy menstrual bleeding | Q103, Y113 | O00-O08*, O60-O69*, O70-O75*,N92, N95 |
| Knee arthroscopy with osteoarthritis | W821 , W822, W823, W828, W829, W851, W852, W853, W858, W859, W831, W832, W833, W834, W835, W836, W837, W838, W839, W841, W842, W843, W844, W861, W879, W901 | M150, M151, M152, M153, M154, M158, M159, M170, M171, M172, M173, M174, M175, M179 |
| Injection for nonspecific low back pain without sciatica | A521 , A522 , A528 , A529 , A577 , A735 , V544 , Z676, Z675, Z993 | M518 , M519 , M545 , M549 |
| Breast reduction | B311 | Z853*, D051*, D059*, D486* |
| Grommets | D151, D289 | H652, H653, H661, H662, H663 , H664, H669 |
| Tonsillectomy | F341, F342, F343, F344, F345, F346, F347, F348, F349, F361 | G470, G471, G472, G473, G474, G478, G479, J36X |
| Haemorrhoid surgery | H511, H512, H513, H518, H519 |  |
| Hysterectomy for heavy bleeding | Q072, Q074, Q078, Q079, Q082, Q088, Q089 | O00-O08*, O60-O69*, O70-O75* |
| Chalazia removal | C121, C122, C124, C191, C198 | H001 |
| Shoulder decompression | O291 | M2551, M754 |
| Carpal tunnel syndrome release | A651, A659 | G560 |
| Dupuytren’s contracture release | T521, T522, T525, T526, T541, T561 | M720 |
| Ganglion excision | T591, T592, T598, T599, T601, T602, T608, T609 | M674 |
| Trigger finger release | T691, T692, T698, T699, T701, T702, T711 , T718, T719, T723, T728, T729, Z894, Z895, Z896, Z897 | M653, M6530, M6531, M6532, M6533, M6534, M6535, M6536, M6537, M6538, M6539 |
| Varicose vein surgery | L832 , L838 , L839 , L841 , L842 , L843 , L844 , L845 , L846 , L848 , L849 , L851 , L852 , L853 , L858 , L859 , L861 , L862 , L868 , L869 , L871 , L872 , L873 , L874 , L875 , L876 , L877 , L878 , L879 , L881 , L882 , L883 , L888 , L889 | I800 , I801 , I802 , I803 , I808 , I809 , I830 , I831 , I832 , I839 |

*Source: NHS England*^1^ **Exclude records whereby primary diagnosis code contains this ICD-10 code*

### Table 2: Privately funded Evidence Based Interventions (EBI) procedures in private hospitals in before and after application of diagnostic code inclusion criteria ^a^

|  | 2017/18 | | 2018/19 | | 2019/20 | |
| --- | --- | --- | --- | --- | --- | --- |
|  | Before | After | Before | After | Before | After |
| Category 1 Procedures | | | | | | |
| Surgical intervention for snoring (not obstructive sleep apnoea) | 67 | 47 | 52 | 35 | 58 | 45 |
| Dilatation & curettage for heavy menstrual bleeding | 65 | 60 | 65 | 57 | 79 | 75 |
| Knee arthroscopy with osteoarthritis | 18,734 | 220 | 17,276 | 339 | 17,223 | 424 |
| Injection for nonspecific low back pain without sciatica | 24,311 | 8,645 | 22,769 | 8,540 | 26,907 | 9,276 |
| Total | 43,177 | 8,972 | 40,162 | 8,971 | 44,267 | 9,820 |
| Category 2 Procedures | | | | | | |
| Breast reduction | 2,801 | 2,630 | 2,879 | 2,701 | 3,068 | 2,852 |
| Grommets | 2,781 | 355 | 2,517 | 329 | 2,712 | 380 |
| Tonsillectomy | 4,717 | 4,051 | 4,794 | 3,945 | 5,079 | 4,007 |
| Haemorrhoid surgery | 1,483 | 1,399 | 1,411 | 1,356 | 1,418 | 1,359 |
| Hysterectomy for heavy bleeding | 3,350 | 2,910 | 3,321 | 2,820 | 3,387 | 2,906 |
| Chalazia removal | 966 | 134 | 982 | 137 | 1,101 | 206 |
| Shoulder decompression | 6,261 | 2,867 | 5,100 | 2,446 | 4,356 | 2,563 |
| Carpal tunnel syndrome release | 3,957 | 2,960 | 3,385 | 2,988 | 4,120 | 3,861 |
| Dupuytren’s contracture release | 1,298 | 869 | 1,398 | 1,056 | 1,365 | 1,223 |
| Ganglion excision | 1,036 | 623 | 891 | 728 | 940 | 849 |
| Trigger finger release | 1,715 | 670 | 1,799 | 801 | 1,926 | 868 |
| Varicose vein surgery | 11,078 | 9,017 | 8,579 | 7,082 | 8,196 | 7,665 |
| Total | 41,443 | 28,485 | 37,056 | 26,389 | 37,668 | 28,739 |
| All EBI Procedures | | | | | | |
| Total | 84,620 | 37,457 | 77,218 | 35,360 | 81,935 | 38,559 |

*^a^These volumes reflect the number of hospital spells identified before and after the application of inclusion criteria for EBI procedures based on International Classification of Diseases 10^th^ Revision (ICD-10) codes developed to reflect instances of low value care.* March has been removed from the above data for all financial years to account for the influence of the emergence of the COVID-19 pandemic.

### Table 3: Completeness of dominant diagnosis coding for privately funded Evidence Based Interventions (EBI) procedures in private hospitals in England 2017/18 to 2019/20 ^a^

|  | 2017/18 | 2018/19 | 2019/20 |
| --- | --- | --- | --- |
| Category 1 Procedures | | | |
| Surgical intervention for snoring (not OSA) | 98.51%  (95.53,101.49%) | 100% | 98.28%  (94.82,101.73%) |
| Dilatation & curettage for heavy menstrual bleeding | 98.46%  (95.39, 101.54%) | 96.92%  (92.61,101.24%) | 97.47%  93.93,101.01%) |
| Knee arthroscopy with osteoarthritis | 98.38%  (98.20,98.56%) | 97.77%  (97.54, 97.99%) | 96.52%  (96.25,96.80%) |
| Injection for nonspecific low back pain without sciatica | 98.84%  (98.70,98.97%) | 98.06%  (97.88,98.24%) | 96.95%  (96.75,97.16%) |
| Total | 98.64%  (98.53,98.75%) | 97.93%  (97.79,98.07%) | 96.79%  (96.62,96.95%) |
| Category 2 Procedures | | | |
| Breast reduction | 96.61%  (95.94,97.28%) | 95.17%  (94.39,95.96%) | 95.83%  (95.12,96.54%) |
| Grommets | 98.99%  (98.62,99.36%) | 99.60%  (99.36,99.85%) | 99.74%  (99.55,99.93%) |
| Tonsillectomy | 98.56%  (98.22,98.90%) | 99.52%  (99.32, 99.72%) | 99.84%  (99.73,99.95%) |
| Haemorrhoid surgery | 98.04 %  (97.34,98.75%) | 99.86%  (99.66,100.06%) | 99.58%  (99.24,99.91%) |
| Hysterectomy for heavy bleeding | 98.84%  (98.47,99.20%) | 99.40%  (99.13,99.66%) | 99.56%  (99.33,99.78%) |
| Chalazia removal | 97.52%  (96.53,98.50%) | 98.68 %  (97.96,99.39%) | 98.46%  (97.73,99.19%) |
| Shoulder decompression | 99.30%  ( 99.09,99.50%) | 98.20%  (97.83,98.56%) | 95.02%  (94.37,95.66%) |
| Carpal tunnel syndrome release | 98.99%  (98.68,99.31%) | 99.35%  (99.08,99.62%) | 99.42%  (99.19,99.65%) |
| Dupuytren’s contracture release | 98.84 %  (98.26,99.43%) | 99.28%  (98.84,99.73%) | 99.34%  (98.91,99.77%) |
| Ganglion excision | 99.13%  (98.57, 99.70%) | 99.44%  (98.94,99.93%) | 99.26%  (98.70,99.81%) |
| Trigger finger release | 99.24%  (98.83,99.65%) | 99.11%  (98.68,99.55%) | 98.55%  (98.01,99.08%) |
| Varicose vein surgery | 98.27%  (98.02,98.51%) | 98.94 %  (98.72,99.16%) | 99.94%  (99.89,99.99%) |
| Total | 98.56%  (98.45,98.68%) | 98.80%  (98.69,98.92%) | 98.75%  (98.64,98.86%) |
| All EBI Procedures | | | |
| Total | 98.60%  (98.5266,98.68%) | 98.35%  (98.26,98.44%) | 97.69%  (97.59, 97.79%) |

^a^These percentages reflect the proportion of hospital spells that contain codes for dominant diagnosis for each EBI procedure prior to the application of the inclusion criteria based on International Classification of Diseases 10^th^ Revision (ICD-10) codes developed to reflect instances of low value care. March has been removed from the above data for all financial years to account for the influence of the emergence of the COVID-19 pandemic.

## Regression Model Equation

The regression model used at both the hospital and local healthcare market level of analysis is below:

1. $\Delta Y_{xt}=\beta_{0}+\beta_{1}\Delta{PFlowvalue}_{xt}+\beta_{2}Z_{xt-1}+\varepsilon_{xt}$

${\Delta Y}_{xt}$ is the dependent variable, the change in volume of privately funded procedures undertaken by hospital or local healthcare market,$x$*,* for each month, *t*, in 2019/20 compared to the same month in 2018/19. ${\Delta PFlowvalue}_{it}$ is the change in volume of publicly funded procedures by the same hospital or local healthcare market,$x$, for each month, *t*, in 2019/20 compared to the same month in 2018/19. March was removed from the data to account for the influence of the emergence of the COVID-19 pandemic. $Z_{xt-1}$is a number of controls reflecting baseline characteristics for each hospital, such as total volume of procedures, region, and aggregate patient characteristics (age, gender, charlson index, and IMD score). The model was run using fixed effects to difference out all time-invariant hospital characteristics out of the equation.

## Assumption and Model Specification tests

### Table 4: Multicollinearity test for association between NHS and privately funded monthly volume change between 2019/20 and 2018/19 for Evidence Based Interventions (EBI) procedures^a^

|  | (Total) | (Category 1) | (Category 2) | (Total) | (Category 1) | (Category 2) |
| --- | --- | --- | --- | --- | --- | --- |
|  | Private  Hospital | Private Hospital | Private Hospital | Local healthcare  market | Local healthcare  market | Local healthcare  market |
| ∆ NHS volume | 1.02 | 1.17 | 1.01 | 1.01 | 1.06 | 1.00 |
|  |  |  |  |  |  |  |
| Gender | 1.04 | 1.07 | 1.05 | 1.03 | 1.01 | 1.04 |
|  |  |  |  |  |  |  |
| CCI | 1.11 | 1.08 | 1.08 | 1.13 | 1.05 | 1.11 |
|  |  |  |  |  |  |  |
| Age | 1.11 | 1.14 | 1.10 | 1.16 | 1.04 | 1.22 |
|  |  |  |  |  |  |  |
| IMD | 1.06 | 1.02 | 1.05 | 1.03 | 1.03 | 1.03 |
|  |  |  |  |  |  |  |
| Total volume | 1.04 | 1.17 | 1.01 | 1.05 | 1.07 | 1.11 |

CCI: Charlson Comorbidty Index, IMD: Index of Multiple Deprivation

^a^The multicollinearity test used is the inverse of the variance inflation factor for each independent variable, a VIF > 10 is understood as warranting investigation. Further information is available here:

Belsley, D. A., E. Kuh, and R. E. Welsch. 1980. Regression Diagnostics: Identifying Influential Data and Sources of Collinearity. New York: Wiley.^2^

### Table 5: Model specification tests for association between NHS and privately funded monthly volume change for Evidence Based Interventions (EBI) procedures

|  | (Total) | (Category 1) | (Category 2) | (Total) | (Category 1) | (Category 2) |
| --- | --- | --- | --- | --- | --- | --- |
|  | Private  Hospital | Private Hospital | Private Hospital | Local healthcare  market | Local healthcare  market | Local healthcare  market |
| Hausman test for fixed versus random effects***^a^*** | 186.75  p = 0.000 | 51.50  P=0.000 | 159.72  P=0.000 | 479.95  P=0.000 | 296.85  P=0.000 | 322.73  P=0.000 |

CCI: Charlson Comorbidty Index, IMD: Index of Multiple Deprivation

^a^ The Hausman test establishes whether fixed rather than random effects is the correct model specification for individual-level effects. A significant p-value implies that fixed effects is the correct model specification.

Further information is available here:

Hausman, J. A. 1978. Specification tests in econometrics. Econometrica 46: 1251–1271.^3^

### Figure 1: Scatter plot graph of observations for dependent and independent variables (Hospital analysis)


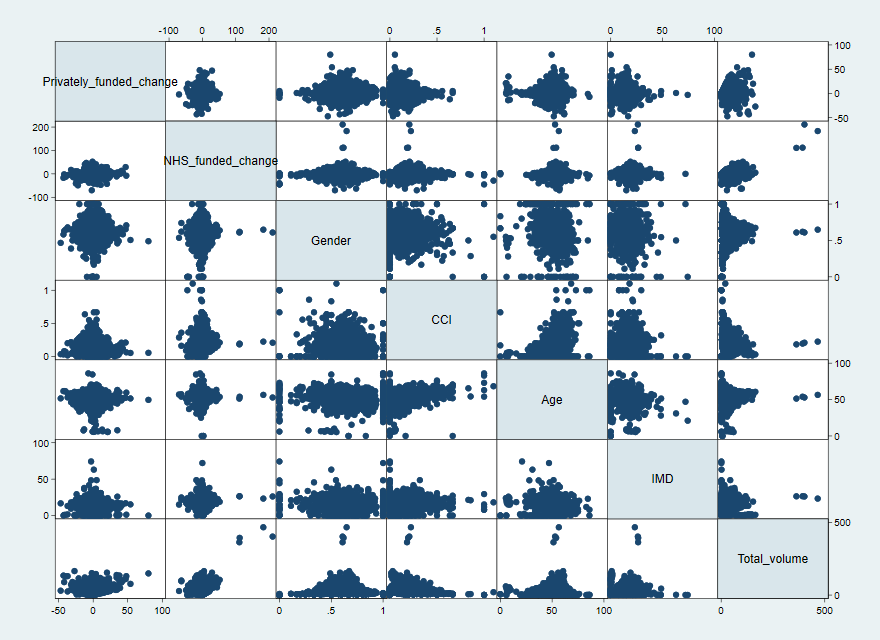


CCI: Charlson Comorbidty Index, IMD: Index of Multiple Deprivation

### Figure 2: Scatter plot graph of observations for dependent and independent variables (Local healthcare market analysis)


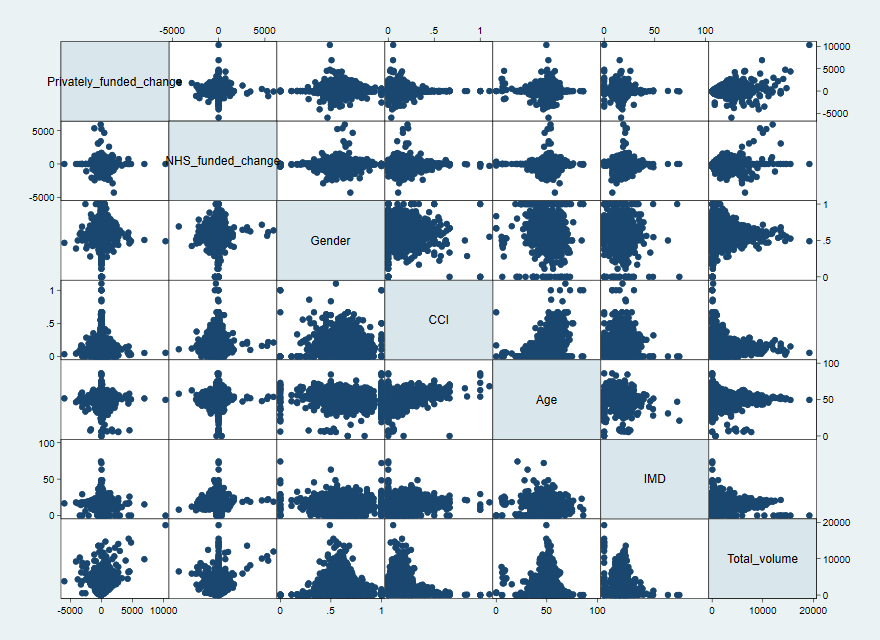


CCI: Charlson Comorbidty Index, IMD: Index of Multiple Deprivation

# Supplementary Results

### Table 6: Association between NHS and Private monthly volume change between 2019/20 and 2017/18 ^a^

|  | (Total) | (Category 1) | (Category 2) | (Total) | (Category 1) | (Category 2) |
| --- | --- | --- | --- | --- | --- | --- |
|  | Private  Hospital | Private Hospital | Private Hospital | Local healthcare  market | Local healthcare  market | Local healthcare  market |
| ∆ NHS volume | -0.15*** | -0.07* | -0.17*** | -0.29*** | -0.43* | -0.28*** |
|  | (0.21, -0.09) | (-0.13, -0.00) | (-0.22, -0.11) | (-0.42, -0.15) | (-0.84, -0.02) | (-0.38, -0.19) |
| Gender | -2.75 | 0.60 | -0.37 | 12.81 | 10.33 | 40.88 |
|  | (-5.74, 0.24) | (-2.02, 3.22) | (-2.94, 2.21) | (-55.35, 80.97) | (-46.08, 66.74) | (-31.94, 113.70) |
| CCI | -3.10 | -3.71** | -2.23 | -32.12 | -15.55 | -75.70* |
|  | (-6.56, 0.36) | (-6.06, -1.37) | (-5.12, 0.65) | (-163.85, 99.82) | (-79.75, 48.65) | (-150.08, -1.31) |
| Age | -0.05 | -0.03 | -0.08* | -1.65 | -0.70 | -2.82* |
|  | (-0.14, 0.03) | (-0.14, 0.08) | (-0.15, -0.00) | (-5.51, 2.20) | (-2.63, 1.24) | (-5.47, -0.17) |
| IMD | -0.06 | -0.11* | -0.06 | 1.19 | -0.03 | -0.33 |
|  | (-0.16, 0.05) | (-0.21, -0.01) | (-0.15, 0.04) | (-1.88, 4.26) | (-1.65, 1.58) | (-3.75, 3.09) |
| Total volume | 0.50*** | 0.46*** | 0.51*** | 0.68*** | 0.89*** | 0.67*** |
|  | (0.42, 0.58) | (0.34, 0.58) | (0.43, 0.58) | (0.53, 0.83) | (0.55, 1.22) | (0.52, 0.83) |
| Constant | -12.41*** | -5.39 | -9.06*** | -739.48*** | -473.43*** | -457.65*** |
|  | (-18.54, -6.29) | (-12.47, 1.70) | (-13.99, -4.14) | (-937.79, -541.17) | (-669.08, -277.79) | (-670.52, -244.77) |
| Observations | 1352 | 349 | 1305 | 1895 | 994 | 1873 |
| Adjusted R^2^ | 0.282 | 0.241 | 0.292 | 0.304 | 0.265 | 0.367 |
| Number of unit of observations | 141 | 64 | 139 | 209 | 158 | 205 |

95% Confidence intervals in parentheses, CCI: Charlson Comorbidty Index, IMD: Index of Multiple Deprivation

*** p<0.001, ** p<0.01, * p<0.05 ^a^ March has been removed from the above data for all financial years to account for the influence of the emergence of the COVID-19 pandemic.

### Table 7: Volumes of NHS, insured, and self-pay funded Evidence Based Interventions (EBI) procedures in private hospitals in England 2017/18 to 2019/20 ^a^

|  | 2017/18 | | | 2018/19 | | | 2019/20 | | |
| --- | --- | --- | --- | --- | --- | --- | --- | --- | --- |
| Procedure | Insured | Self  Pay | NHS Funded | Insured | Self  Pay | NHS Funded | Insured | Self  Pay | NHS Funded |
| Category 1 Procedures | | | | | | | | | |
| Intervention for snoring (not OSA) | 27 | 20 | 56 | 20 | 15 | 51 | 29 | 12 | 40 |
| Dilatation & curettage for heavy menstrual bleeding | 52 | 8 | 88 | 47 | 8 | 40 | 59 | * | 52 |
| Knee arthroscopy with osteoarthritis | 188 | 32 | 1,349 | 281 | 54 | 871 | 325 | 63 | 763 |
| Injection for nonspecific low back pain without sciatica | 6,763 | 1,881 | 7,860 | 6,560 | 1,853 | 7,470 | 6,525 | 2,095 | 6,546 |
| Total | 7030 | 1941 | 9353 | 6908 | 1930 | 8432 | 6938 | 2177 | 7401 |
| Category 2 Procedures | | | | | | | | | |
| Breast reduction | 461 | 2,168 | * | 407 | 2,281 | 9 | 442 | 2,365 | * |
| Grommets | 265 | 90 | 221 | 237 | 90 | 230 | 280 | 85 | 227 |
| Tonsillectomy | 3,373 | 677 | 1,750 | 3,064 | 779 | 1,688 | 2,711 | 833 | 1,264 |
| Haemorrhoid surgery | 1,186 | 212 | 1,680 | 1,092 | 227 | 1,655 | 989 | 207 | 1,611 |
| Hysterectomy for heavy bleeding | 2,387 | 523 | 1,793 | 2,287 | 506 | 1,728 | 2,242 | 552 | 1,693 |
| Chalazia removal | 103 | 31 | 205 | 99 | 36 | 188 | 161 | 39 | 187 |
| Shoulder decompression | 2,598 | 269 | 5,309 | 2,164 | 242 | 3,795 | 2,259 | 218 | 2,613 |
| Carpal tunnel syndrome release | 2,219 | 741 | 11,384 | 2,159 | 792 | 10,307 | 2,667 | 1,058 | 9,968 |
| Dupuytren’s contracture release | 707 | 162 | 3,625 | 858 | 191 | 3,710 | 943 | 227 | 3,583 |
| Ganglion excision | 542 | 81 | 1,926 | 623 | 93 | 1,537 | 722 | 85 | 1,522 |
| Trigger finger release | 571 | 99 | 2,323 | 679 | 115 | 2,248 | 701 | 137 | 2,061 |
| Varicose vein surgery | 4,522 | 4,495 | 1,922 | 4,508 | 2,517 | 1,804 | 4,711 | 2,719 | 1,563 |
| Total | 18,934 | 9,548 | 32,144 | 18,177 | 7,869 | 28,899 | 18828 | 8525 | 26,298 |

*PHIN applies a policy of small number suppression for any activities levels <8, OSA=Obstructive sleep apnoea. ^a^ There are slight discrepancies between total volumes of privately funded care in Table 2 of the main manuscript and this table as coding for patients accessing care through self-pay or insured mechanisms was only available for 94.58% of patients in 2019/20, 98.65% of patients in 2018/19, and 99.99% in 2017/18. March has been removed from the above data for all financial years to account for the influence of the emergence of the COVID-19 pandemic.

### Table 8: Association between NHS and privately funded monthly volume change between 2019/20 and 2018/19 (Insurance analysis) ^a^

|  | (Total) | (Category 1) | (Category 2) | (Total) | (Category 1) | (Category 2) |
| --- | --- | --- | --- | --- | --- | --- |
|  | Private  Hospital | Private Hospital | Private Hospital | Local healthcare  market | Local healthcare  market | Local healthcare  market |
| ∆ NHS volume | -0.13*** | -0.16** | -0.15*** | -0.25** | -0.09 | -0.20*** |
|  | (-0.19, -0.07) | (-0.27, -0.05) | (-0.21, -0.09) | (-0.41, -0.10) | (-0.26, 0.07) | (-0.28, -0.12) |
| Gender | 1.72 | -1.37 | 1.94 | -15.58 | -2.34 | -27.63 |
|  | (-1.01, 4.46) | (-4.17, 1.42) | (-0.41, 4.30) | (-91.79, 60.64) | (-36.10, 31.41) | (-104.67, 49.40) |
| CCI | -0.05 | -4.34** | -0.61 | -34.09 | -35.18** | -33.50 |
|  | (-3.52, 3.41) | (-7.20, -1.48) | (-3.20, 1.97) | (-105.91, 37.73) | (-60.74, -9.62) | (-97.62, 30.61) |
| Age | -0.06 | -0.06 | -0.08* | -2.34* | -0.21 | -2.29** |
|  | (-0.15, 0.02) | (-0.14, 0.02) | (0.15, -0.01) | (-4.20, -0.48) | (-1.63, 1.20) | (-3.99, -0.59) |
| IMD | -0.17* | -0.06 | -0.08 | -2.79 | -0.45 | -1.49 |
|  | (-0.30, -0.04) | (-0.17, 0.05) | (-0.18, 0.02) | (-6.02, 0.44) | (-1.76, 0.86) | (-4.26, 1.27) |
| Total volume | 0.38*** | 0.47*** | 0.41*** | 0.66*** | 0.81** | 0.53*** |
|  | (0.32, 0.45) | (0.33, 0.62) | (0.35, 0.46) | (0.34, 0.99) | (0.21, 1.40) | (0.43, 0.64) |
| Constant | -7.38* | -2.58 | -5.71* | -456.95** | -352.95** | -213.66** |
|  | (-13.32, -1.44) | (-7.90, 2.74) | (-10.76, -0.67) | (-793.35, -120.55) | (-617.60, -88.29) | (-360.93, -66.39) |
| Observations | 1279 | 315 | 1227 | 1979 | 1043 | 1956 |
| Adjusted R^2^ | 0.161 | 0.251 | 0.159 | 0.383 | 0.482 | 0.236 |
| No. of hospitals | 140 | 63 | 138 | 198 | 154 | 196 |

95% Confidence intervals in parentheses, CCI: Charlson Comorbidty Index, IMD: Index of Multiple Deprivation

*** p<0.001, ** p<0.01, * p<0.05 ^a^ March has been removed from the above data for all financial years to account for the influence of the emergence of the COVID-19 pandemic.

### Table 9: Association between NHS and privately funded monthly volume change between 2019/20 and 2018/19 (Self pay analysis) ^a^

|  | (Total) | (Category 1) | (Category 2) | (Total) | (Category 1) | (Category 2) |
| --- | --- | --- | --- | --- | --- | --- |
|  | Private  Hospital | Private  Hospital | Private Hospital | Local healthcare  market | Local healthcare  market | Local healthcare  market |
| ∆ NHS volume | -0.09*** | -0.17*** | -0.11*** | -0.10** | -0.02 | -0.15*** |
|  | (-0.14,-0.05) | (-0.27,-0.08) | (-0.16, -0.06) | (-0.18, -0.03) | (-0.06, 0.03) | (-0.21, -0.09) |
| Gender | 3.08** | -2.40* | 3.39*** | 20.28 | -16.27 | 11.22 |
|  | (1.21, 4.96) | (-4.47, -0.34) | (1.65, 5.14) | (-18.12, 58.68) | (-35.54, 2.99) | (-24.18, 46.61) |
| CCI | -2.09* | -1.89 | -1.26 | -6.52 | -7.03 | -6.33 |
|  | (-3.87, -0.30) | (-4.19, 0.42) | (-2.71, 0.19) | (-44.21, 31.17) | (-17.97, 3.92) | (-34.51, 21.85) |
| Age | 0.02 | 0.07* | -0.01 | -0.01 | 0.38 | -0.28 |
|  | (-0.03, 0.08) | (0.00, 0.13) | (-0.06, 0.04) | (-0.92, 0.91) | (-0.06, 0.83) | (-0.92, 0.37) |
| IMD | -0.06 | -0.04 | -0.06* | -1.18 | -0.06 | -2.07 |
|  | (-0.14, 0.01) | (-0.14, 0.05) | (-0.12, -0.00) | (-3.52, 0.89) | (-0.52, 0.41) | (-4.25, 0.11) |
| Total volume | 0.24*** | 0.36*** | 0.28*** | 0.23* | 0.12 | 0.30*** |
|  | (0.17, 0.32) | (0.25, 0.48) | (0.20, 0.35) | (0.05, 0.40) | (-0.05, 0.30) | (0.17, 0.43) |
| Constant | -8.08*** | -6.28* | -6.16** | -128.64* | -32.55 | -110.04** |
|  | (-12.45, -3.70) | (-11.03, -1.52) | (-10.09, 2.22) | (-235.92, -21.36) | (-94.02, 28.91) | (-176.22, -43.87) |
| Observations | 1125 | 203 | 1074 | 1992 | 874 | 1956 |
| Adjusted R^2^ | 0.127 | 0.255 | 0.147 | 0.117 | 0.081 | 0.124 |
| No. of hospitals | 139 | 48 | 135 | 208 | 141 | 203 |

95% Confidence intervals in parentheses, CCI: Charlson Comorbidty Index, IMD: Index of Multiple Deprivation

*** p<0.001, ** p<0.01, * p<0.05 ^a^ March has been removed from the above data for all financial years to account for the influence of the emergence of the COVID-19 pandemic.

### Table 10: Association between NHS and privately funded monthly volume change between 2019/20 and 2017/18 (Insurance analysis) ^a^

|  | (Total) | (Category 1) | (Category 2) | (Total) | (Category 1) | (Category 2) |
| --- | --- | --- | --- | --- | --- | --- |
|  | Private  Hospital | Private Hospital | Private Hospital | Local healthcare  market | Local healthcare  market | Local healthcare  market |
| ∆ NHS volume | -0.12*** | -0.09** | -0.13*** | -0.24*** | -0.35* | -0.26 |
|  | (-0.17, -0.06) | (-0.16, -0.02) | (-0.18, -0.08) | (-0.36, -0.13) | (-0.69, -0.02) | (-0.35,0.17) |
| Gender | -1.40 | 0.19 | -0.34 | 2.61 | 21.02 | 28.49 |
|  | (-3.80, 1.00) | (-1.75, 2.13) | (-2.36, 1.67) | (-67.80, 73.03) | (-15.97, 58.00) | (-42.47, 99.45) |
| CCI | -3.09* | -2.63 | -2.19 | -68.88 | -16.39 | -73.81* |
|  | (-5.80, -0.38) | (-5.40, 0.14) | (-4.61, 0.23) | (-145.20, 7.44) | (-47.42, 14.63) | (-143.29, -4.33) |
| Age | -0.07 | -0.05 | -0.08** | -0.94 | -0.87 | -0.98 |
|  | (-0.14, 0.01) | (-0.15, 0.05) | (-0.13, -0.02) | (-3.14, 1.27) | (-1.92, 0.21) | (-2.98, 1.02) |
| IMD | -0.08 | -0.09 | -0.03 | -1.61 | -1.02 | -0.74 |
|  | (-0.19, 0.03) | (-0.19, 0.01) | (-0.12, 0.06) | (-4.42, 1.21) | (-2.17, 0.13) | (-4.15, 2.67) |
| Total volume | 0.40*** | 0.44*** | 0.41*** | 0.60*** | 0.72*** | 0.61*** |
|  | (0.33, 0.48) | (0.33, 0.56) | (0.35, 0.48) | (0.44, 0.76) | (0.36, 1.08) | (0.47, 0.76) |
| Constant | -7.22* | -3.42 | -5.53** | -497.23*** | 264.72*** | -401.42*** |
|  | (-12.74, -1.71) | (-9.45, 2.61) | (-9.38, -1.68) | (-654.22, -340.24) | (-428.26, -101.18) | (-540.07, -262.77) |
| Observations | 1301 | 316 | 1244 | 1788 | 913 | 1762 |
| Adjusted R^2^ | 0.218 | 0.225 | 0.233 | 0.322 | 0.351 | 0.325 |
| No. of hospitals | 137 | 58 | 134 | 199 | 154 | 197 |

95% Confidence intervals in parentheses, CCI: Charlson Comorbidty Index, IMD: Index of Multiple Deprivation

*** p<0.001, ** p<0.01, * p<0.05 ^a^ March has been removed from the above data for all financial years to account for the influence of the emergence of the COVID-19 pandemic.

### Table 11: Association between NHS and privately funded monthly volume change between 2019/20 and 2017/18 (Self pay analysis) ^a^

|  | (Total) | (Category 1) | (Category 2) | (Total) | (Category 1) | (Category 2) |
| --- | --- | --- | --- | --- | --- | --- |
|  | Private  Hospital | Private Hospital | Private Hospital | Local healthcare  market | Local healthcare  market | Local healthcare  market |
| ∆ NHS volume | -0.10*** | -0.03 | -0.11*** | -0.12*** | -0.06 | -0.12*** |
|  | (-0.14, -0.07) | (-0.10, 0.04) | (-0.14, -0.07) | (-0.17, 0.06) | (-0.22, 0.09) | (-0.17, -0.06) |
| Gender | 0.25 | -0.35 | 1.14 | -0.76 | -17.45 | 3.87 |
|  | (-1.52, 2.01) | (-2.50, 1.79) | (-0.59, 2.88) | (-27.51, 26.00) | (-33.87, -1.03) | (-22.71, 30.45) |
| CCI | -0.86 | -0.36 | -0.58 | -11.12 | -2.67 | 11.28 |
|  | (-2.73, 1.02) | (-2.27, 1.54) | (-2.36, 1.20) | (-45.32, 23.08) | (-20.20, 14.85) | (-23.14, 45.71) |
| Age | 0.01 | 0.02 | 0.01 | 0.36 | -0.30 | -0.23 |
|  | (-0.04, 0.06) | (-0.04, 0.08) | (-0.04, 0.05) | (-0.56, 1.29) | (-0.90, 0.29) | (-1.01, 0.56) |
| IMD | -0.04 | -0.03 | -0.09*** | -0.63 | -0.23 | -1.52* |
|  | (-0.10, 0.01) | (-0.10, 0.03) | (-0.13, -0.04) | (-1.78, 0.52) | (-0.84, 0.37) | (-2.85, -0.18) |
| Total volume | 0.27*** | 0.25** | 0.28*** | 0.25*** | 0.16 | 0.26*** |
|  | (0.21, 0.33) | (0.09, 0.41) | (0.21, 0.35) | (0.13, 0.38) | (-0.07, 0.38) | (0.13, 0.39) |
| Constant | -6.79*** | -3.23 | -5.30** | -152.71** | 2.86 | -92.77* |
|  | (-10.47, -3.12) | (-8.16, 1.70) | (-8.47, -2.14) | (-254.36, -51.06) | (-77.61, 83.33) | (-172.68, -12.87) |
| Observations | 1062 | 200 | 1019 | 1792 | 791 | 1756 |
| Adjusted R^2^ | 0.157 | 0.137 | 0.166 | 0.176 | 0.084 | 0.159 |
| No. of hospitals | 133 | 44 | 56 | 208 | 139 | 185 |

95% Confidence intervals in parentheses, CCI: Charlson Comorbidty Index, IMD: Index of Multiple Deprivation

*** p<0.001, ** p<0.01, * p<0.05 ^a^ March has been removed from the above data for all financial years to account for the influence of the emergence of the COVID-19 pandemic.

### Table 12: Association between NHS and privately funded monthly volume change between 2019/20 and 2018/19 for individual Evidence Based Interventions (EBI) procedures ^a^

|  | ∆ Total private volume | ∆ Insured volume | ∆ Self-pay volume | ∆ Total private volume | ∆ Insured volume | ∆ Self-pay volume |
| --- | --- | --- | --- | --- | --- | --- |
|  | (Private Hospitals) | (Private Hospitals) | (Private Hospitals) | (Local healthcare market) | (Local healthcare market) | (Local healthcare market) |
| **Category 1 Procedures** |  |  |  |  |  |  |
| Surgical intervention for snoring (not OSA) | - | - | - | - | - | - |
|  |  |  |  |  |  |  |
| Dilatation & curettage for heavy menstrual bleeding | - | - | - | - | - | - |
|  |  |  |  |  |  |  |
| Knee arthroscopy with osteoarthritis | 0.02 | - | - | -0.11 | -0.00 | - |
|  | (-1.74,1.78) |  |  | (-0.32,0.11) | (-0.03,0.02) |  |
| Injection for nonspecific low back pain without sciatica | -0.08* | -0.09** | -0.02 | -0.54* | -0.47* | -0.07 |
|  | (-0.14,-0.02) | (-0.15,-0.03) | (-0.08,0.04) | (-1.02,-0.07) | (-0.89,-0.05) | (-0.24,0.09) |
| **Category 2 Procedures** |  |  |  |  |  |  |
| Breast reduction | - | - | - | - | - | - |
|  |  |  |  |  |  |  |
| Grommets | - | - | - | -0.37* | -0.37* | - |
|  |  |  |  | (-0.70,-0.04) | (-0.69,-0.06) |  |
| Tonsillectomy | -0.31*** | -0.13* | -0.16 | -0.30* | -0.16 | -0.28*** |
|  | (-0.39,-0.23) | (-0.24,-0.03) | (-0.38,0.06) | (-0.58,-0.03) | (-0.47,0.15) | (-0.37,-0.18) |
| Haemorrhoid surgery | -0.13 | -0.13 | - | -0.41*** | -0.25** | -0.01 |
|  | (-0.30,0.04) | (-0.29,0.03) |  | (-0.63,-0.20) | (-0.41,-0.08) | (-0.02,0.00) |
| Hysterectomy for heavy bleeding | -0.25** | -0.22** | -0.37* | -0.49*** | -0.42*** | -0.06* |
|  | (-0.40,-0.10) | (-0.38,-0.06) | (-0.68,-0.06) | (-0.67,-0.30) | (-0.60,-0.24) | (-0.12,-0.00) |
| Chalazia removal | - | - | - | - | - | - |
|  |  |  |  |  |  |  |
| Shoulder decompression | -0.09 | -0.05 | 0.28*** | -0.02 | -0.01 | -0.01 |
|  | (-0.22,0.05) | (-0.18,0.09) | (0.26,0.30) | (-0.31,0.26) | (-0.26,0.24) | (-0.02,0.01) |
| Carpal tunnel syndrome release | -0.16** | -0.10* | -0.08** | -0.18*** | -0.13* | -0.01 |
|  | (-0.26,-0.05) | (-0.18,-0.02) | (-0.15,-0.02) | (-0.28,-0.08) | (-0.23,-0.02) | (0.02, 0.00) |
| Dupuytren’s contracture release | -0.14* | -0.13 | - | -0.07* | -0.05* | - |
|  | (-0.25,-0.03) | (-0.29,0.02) |  | (-0.12,-0.02) | (-0.09,-0.01) |  |
| Ganglion excision | -0.25 | -0.00 | - | -0.09 | -0.03 | - |
|  | (-0.53,0.02) | (-0.39,0.38) |  | (-0.25,0.06) | (-0.07,0.02) |  |
| Trigger finger release | -0.49** | -0.46* | - | -0.30*** | -0.15* | - |
|  | (-0.81,-0.17) | (-0.81,-0.11) |  | (-0.46,-0.15) | (-0.28,-0.02) |  |
| Varicose vein surgery | -0.20** | -0.20** | -0.18 | -0.34*** | -0.40*** | -0.26*** |
|  | (-0.31,-0.09) | (-0.33,-0.08) | (-0.37,0.02) | (-0.45,-0.22) | (-0.54,-0.26) | (-0.37,-0.15) |

95% Confidence intervals in parentheses, *** p<0.001, ** p<0.01, * p<0.05. – observations in changes in volume for this procedure or financial mechanism were not sufficient to produce co-efficient estimates. ^a^March has been removed from the above data for all financial years to account for the influence of the emergence of the COVID-19 pandemic.

# References

1 NHS England. Evidence-Based Interventions: Guidance for CCGs. 2018. https://www.aomrc.org.uk/ebi/wp-content/uploads/2021/05/ebi-statutory-guidance.pdf (accessed Aug 17, 2022).

2 Belsley DA, Kuh E, Welsch RE. Regression Diagnostics: Identifying Influential Data and Sources of Collinearity. John Wiley & Sons, 2005.

3 Hausman JA. Specification Tests in Econometrics. *Econometrica* 1978; **46**: 1251–71.
